# Marine *Dadabacteria* exhibit genome streamlining and phototrophy-driven niche partitioning

**DOI:** 10.1038/s41396-020-00834-5

**Published:** 2020-11-23

**Authors:** Elaina D. Graham, Benjamin J. Tully

**Affiliations:** 1 Department of Biological Sciences, University of Southern California, Los Angeles, CA USA; 2 Center for Dark Energy Biosphere Investigations, University of Southern California, Los Angeles, CA USA

**Keywords:** Water microbiology, Marine microbiology, Metagenomics, Microbial ecology

## Abstract

The remineralization of organic material via heterotrophy in the marine environment is performed by a diverse and varied group of microorganisms that can specialize in the type of organic material degraded and the niche they occupy. The marine *Dadabacteria* are cosmopolitan in the marine environment and belong to a candidate phylum for which there has not been a comprehensive assessment of the available genomic data to date. Here in, we assess the functional potential of the marine pelagic *Dadabacteria* in comparison to members of the phylum that originate from terrestrial, hydrothermal, and subsurface environments. Our analysis reveals that the marine pelagic *Dadabacteria* have streamlined genomes, corresponding to smaller genome sizes and lower nitrogen content of their DNA and predicted proteome, relative to their phylogenetic counterparts. Collectively, the *Dadabacteria* have the potential to degrade microbial dissolved organic matter, specifically peptidoglycan and phospholipids. The marine *Dadabacteria* belong to two clades with apparent distinct ecological niches in global metagenomic data: a clade with the potential for photoheterotrophy through the use of proteorhodopsin, present predominantly in surface waters up to 100 m depth; and a clade lacking the potential for photoheterotrophy that is more abundant in the deep photic zone.

## Introduction

Heterotrophy in the marine environment is a complex process with many organisms contributing to the remineralization of organic matter. In the surface ocean, ~50% of new organic carbon is remineralized by heterotrophs within the first 100 m [1, 2]. Despite the importance of this process to the overall ocean carbon budget, the specific contributions of the phylogenetically diverse marine bacterioplankton community remain poorly constrained. The metabolic capacity of the community members directly governs the types of organic material that can be degraded in a particular environment [3]. Heterotrophs occupy a spectrum of metabolic diversity and growth strategies [4]. While copiotrophs exploit multiple organic resources and/or undergo rapid growth in response to nutrient availability, oligotrophs specialize in a limited number of resources and dominate in low nutrient environments [5]. Because of the interplay of heterotrophs on this spectrum of metabolic diversity, it is important to understand the role(s) that specific groups play in the degradation of organic matter in the surface ocean.

An evolutionary feature that has been observed among marine oligotrophs is the reduction and simplification of the genome. This evolutionary trajectory has been posited as the theory of genome streamlining, in which organisms that grow in nutrient limited environments undergo selection to reduce cellular demand for specific compounds and nutrients [6]. While originating in the marine environment [7, 8], genome streamlining has been identified in numerous habitats for a variety of microorganisms [9–12]. Streamlined genomes will tend to have smaller genome sizes as a result of increased coding density and a decreased number of paralogs/gene duplication events, which overall reduce cellular demand for nutrients [13]. Additionally, in nitrogen-limited environments, streamlined genomes may reduce the contribution of nitrogen to the DNA by decreasing genomic GC content and the proteome through the selection of amino acids with side chains that contain fewer nitrogen atoms [13]. The theory of genome streamlining is an important avenue for understanding microbiology and provides important insights into the evolutionary history and ecological distributions of a microorganism.

Here in, we assess the potential contributions of the *Dadabacteria* to marine heterotrophy. A phylum level group phylogenetically clustered near the phyla *Campylobacteria*, *Aquificota*, and *Deferribacteres*. The *Dadabacteria* (formerly SBR1093) lack a cultured representative and have not been extensively assessed for their potential contributions to biogeochemical cycles though they have been detected in numerous environments. The first *Dadabacteria* genome was reconstructed from industrial activated sludge and reported to possess the capacity for carbon fixation through the 3-hydroxybutyrate/4-hydroxypropionate cycle [14]. Interestingly, multiple *Dadabacteria* metagenome-assembled genomes (MAGs) were reconstructed from the *Tara* Oceans global, marine metagenomic samples, though their exact role in the marine environment was unknown [15–17]. Our analysis reveals that the marine *Dadabacteria* are likely heterotrophic oligotrophs that have undergone genome streamlining with the capacity to degrade microbially derived peptidoglycan as a carbon source with further metabolic diversification between shallow and deep photic zone niches.

## Materials and methods

### Collect, assess and clean genomes, and construct phylogenomic trees

MAGs generated from several studies using the *Tara* Oceans metagenomics dataset were initially identified as *Dadabacteria* based on 16S rRNA phylogeny and 16 concatenated ribosomal proteins (ribosomal proteins L2, L3, L4, L5, L6, L14, L16, L18, L22, L24, S3, S8, S10, S17, and S19) [18]. All *Dadabacteria* metagenome-assembled genomes (MAGs) identified in NCBI (as of August 2019) [19–23] and one *Dadabacteria* genome (formally Candidate Phylum SBR1093) derived from Wang et al. [14] were also included. Genomes reconstructed from Tully et al. [15] and Tully et al. [24] were subjected to manual assessments for quality using the same methodology as in Graham et al. [25]. Briefly, read coverage and DNA compositional data were utilized to bin additional contigs (>5 kb) from the *Tara* Oceans Longhurst province where the original *Dadabacteria* MAG was reconstructed using CONCOCT (v.0.4.1; parameters: -c 800 -I 500) [26]. Bins determined through CONCOCT with overlapping contigs in a *Dadabacteria* MAG were profiled (anvi-profile default parameters), combined (anvi-merge default parameters) and visualized (anvi-interactive default parameters) in anvi’o [27] (v5.0). MAGs were manually refined by removing contigs with incongruent composition metrics or coverage values. Genomes from Delmont et al. [17] were also visualized in anvi’o and manually curated based on composition metrics only. Bin refinement was conducted to minimize contamination estimates and improve genome completion.

*Dadabacteria* MAGs were assessed for quality through the PhyloSanity workflow (default parameters) of the tool MetaSanity [28] (beta version; v1). Estimated completeness, contamination, and strain heterogeneity were determined using CheckM (v1.0.18; lineage_wf default parameters) [29]. The estimated completeness and MAG size were used to calculate an approximate genome size for the complete genome. Additionally, the CheckM Reported Statistics subcommand (checkm qa --tab-table) was used to calculate the coding density. Phylogeny was confirmed using GTDB-Tk (v1.0.0; database ver. 89; classify_wf default parameters) [30]. The GTDB-Tk de novo workflow was used to construct a multiple sequence alignment (MSA) of the *Dadabacteria* MAGs using the bac120 marker set and with f_SZUA-79 set as the outgroup. The full MSA was reduced to include the following lineages related to the *Dadabacteria*: SZUA-79, *Chrysiogenetota*, *Deferribacterota*, *Thermosulfidibacterota*, *Aquificota*, *Camplyobacterota*. The MSA was refined using MUSCLE (v3.8.31, parameter: -refine) [31] and FastTree (v2.1.10, parameters: -lg, -gamma) [32] was used to generate a phylogenetic tree that was visualized using the Interactive Tree of Life (IToL) [33] (Supplementary Data 1).

### Functional annotation

For functional annotation and evidence of genomic streamlining, due to the limited number of available MAGs, all genomes were considered during the analysis. *Dadabacteria* MAGs were assessed for putative metabolic functionality through the FuncSanity workflow of the tool MetaSanity [28] (beta version;v1). All downstream analyses use the putative CDS (coding DNA sequences) as predicted by Prokka (v1.13.3) [34]. Putative CDS were assigned to carbohydrate-active enzyme (CAZy) families based on HMMs (hidden Markov models) from dbCAN (v6) [35] using hmmsearch (v3.1b2; parameter: -T 75) [36]. The output from MetaSanity that combines the CAZy matches for all submitted genomes (MetaSanity output file: combined.cazy) was used to determine the number of CAZy matches per Mbp in each MAG, including a curated selection of glycoside hydrolases (GH) and carbohydrate-binding module (CBM) containing proteins and excluding matches to CAZy subfamily HMMs (e.g., matches to GH13 model were included, while matches to GH13_9 model, etc. were excluded).

CDS were determined to be putative peptidases through hmmsearch (parameter: -T 75) using PFAM [37] HMMs selected to represent the MEROPS families [38]. Putative peptidases were screened for signatures denoting possible extracellular localization using PSORTb (v3.0; parameters: --cutoff 1, --divergent 1, -M 10, -c 70) [39] and SignalP (v4.1; defaults) [40]. First, PSORTb was used to identify all putative peptidases with the localization assignment of “extracellular”, “cellwall”, or “unknown”. For any putative peptidase that had “unknown” localization, if SignalP predicted a transmembrane helix, the peptidase was determined to be putatively extracellular.

Metabolic functions of interest were identified based on the KEGG-Decoder [25] output (v1.0.10) as implemented in MetaSanity (MetaSanity output file: KEGG.final.tsv). As part of this workflow, CDS were assigned to KEGG Ontology (KO) identifiers using KofamScan (v1.2.0) [41] and the accompanying KOfam HMMs. KO annotations were then assigned to a set of manually curated pathways and processes. Additionally, metabolisms of interest, especially those lacking KOfam HMMs, were searched independently and incorporated using KEGG-Expander as implemented in MetaSanity.

Additional databases were used to identify feature of interests within the *Dadabacteria* MAGs. Putative metabolic functions of interest shared between the four phylogenetic clades were identified using eggNOG-mapper [42] (http://eggnog-mapper.embl.de/; default parameters for “Auto adjust per query”) and precomputed eggNOG clusters (v5.0) [43]. antiSMASH (v5.0.0) [44] was used to detect secondary metabolite biosynthetic gene clusters (parameters: --cb-general --cb-knownclusters --cb-subclusters --asf --pfam2go --smcog-trees). Based on matches to the rhodopsin PFAM HMM model (PF01036) performed as part of the KEGG-Decoder analysis, putative rhodopsin CDS were compared to the MicRhoDE database [45] using BLASTP [46] (http://application.sb-roscoff.fr/micrhode/doblast; default parameters for “All Micrhode” option) and assigned to a previously identified clades of rhodopsins based on the highest scoring result (Supplementary Data 2). Additionally, putative rhodopsins were aligned with MUSCLE (parameter: -iter 8) and the 17 amino acid (aa) region that contains the crucial aa for determining function (aa site 97 & 108) and spectral tuning (aa site 105) were categorized based on known rhodopsin relationships (Supplementary Data 3).

### Genomic streamlining

Putative CDS were used to calculate the total number of carbon and nitrogen atoms present in the predicted proteome and the corresponding ratio of each MAG (https://github.com/edgraham/CNratio). For identifying duplicate genes in a MAG, first, all putative CDS in a MAG was compared against each other using DIAMOND BLASTP [47] (parameters: --more-sensitive –max-taget-seqs 300). BLAST matches were filtered using the minbit approach [48], where significant matches were determined based on the relative comparison of bitscore values. Minbit was calculated for protein A compared to protein B, as in Eq. (1),

1 $$\frac{{bitscore\left( {\left[ A \right]\left[ B \right]} \right)}}{{min\left( {bitscore\left( {\left[ A \right]\left[ A \right]} \right),bitscore\left( {\left[ B \right]\left[ B \right]} \right)} \right)}}$$

retaining all BLAST matches ≥0.5. BLAST matches above this threshold were reformatted and clustered using MCL [49] (mcxload parameters: --abc --stream-mirror --stream-neg-log10 -stream-tf ceil(200); mcl default parameters; mcxdump parameter: -icl). All clusters in the mcxdump output were considered to be gene duplication events within the MAG.

### Ecological distribution and environmental correlations

For determining the ecological distribution and environmental correlations, a nonredundant set of MAGs was determined using FastANI [50] (v1.3; parameters: --frag-length 1500) with a representative selected from a cluster of genomes with ≥98.5% average nucleotide identity [51]. Metagenomes derived from bioGEOTRACES [52] (bGT) and *Tara* Oceans [53] were mapped against the nonredundant set of *Dadabacteria* genomes using bowtie2 [54] (v2.3.4.1, parameters: -q, --no-unal), converted from a SAM to BAM file using samtools [55] (v.1.9; view; sort), and filtered using BamM (v1.7.0, parameters: --percentage_id 0.95, --percentage_aln 0.75). featureCounts [56] (v1.5.3, default parameters) implemented through Binsanity-profile [57] (v0.3.3, default parameters) was used to generate read counts for each contig from the filtered BAM files. Read counts were used to calculate the relative fraction of each genome in the sample (Eq. (2)) and determine the reads per kbp of each genome per Mbp of metagenomic sample (RPKM) (Eq. (3)).

2 $$relative\,fraction = \frac{{\# reads\,recruited\,to\,genome}}{{total\,reads\,in\,sample}}$$

3 $$RPKM = \frac{{\# reads\,recruited\,to\,a\,genome \div \left( {genome\,length\,in\,bp \div 1000} \right)}}{{total\,bp\,in\,metagenome \div 1,000.000}}$$

Environmental data were accessed from GEOTRACES Intermediate Data Product 2017 (Version 2) [58] and paired with the corresponding metagenome sample ID. In many cases there were multiple CTD casts associated with a particular station and depth (Supplementary Data 4). The mean value was used in cases where a parameter was measured multiple times at the same depth and station. Environmental data were paired with a metagenome only if the depth was within 1 m of the metagenome. RPKM values for *Dadabacteria* genomes from all samples with available environmental data were used in a canonical correspondence analysis (CCA) in Past4 [59] (v.4.01). Only environmental data that were measured for ≥90% of the samples were used to perform the CCA. RPKM values were normalized (log(*n* + 1)) prior to CCA. Transect plots were made in Ocean Data View (v5.2.1; DIVA Gridding; *Schlitzer, Reiner*, Ocean Data View, https://odv.awi.de, 2020). Bathymetry was pulled from General Bathymetric Chart of the Oceans (GEBCO 2014; 10.1564/PANGAEA.708081).

## Results and discussion

**Fig. 1:**
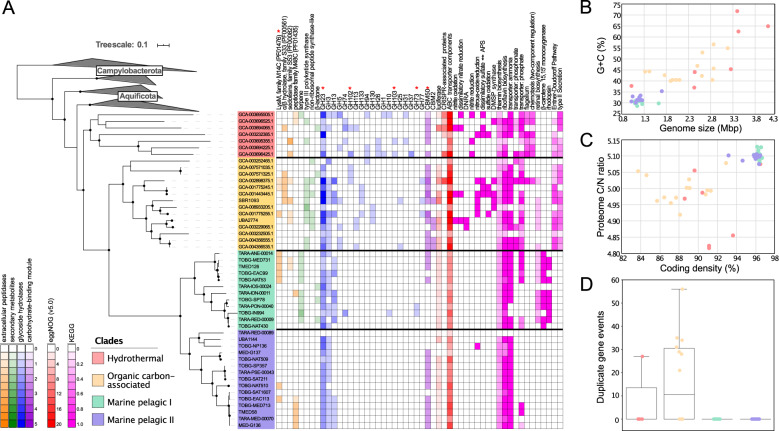
Phylogenomic, functional, and evolutionary relationships amongst the *Dadabacteria*. **a** A phylogenomic tree of the bac120 marker set for the *Dadabacteria* and related phyla and a heatmap displaying functions of interest for each *Dadabacteria* MAG. Bootstrap (1000 resamples) values are scaled proportionally between 0.75 and 1. Putative extracellular peptidase, secondary metabolite, glycoside hydrolase, and carbohydrate-binding module counts are displayed on a scale from 0 to 5. Functions inferred from eggNOG counts are displayed on a scale from 0–20+. Metabolic processes inferred from KEGG are displayed on a scale for 0–1, as a fraction of a particular metabolism detected. MAGs abbreviations: TOBG from Tully et al. [15]; TMED from Tully et al. [24]; TARA from Delmont et al. [17]; MED from López-Pérez et al. [69]; UBA from Parks et al. [16]. **b** A scatter plot of percent G + C (%G + C) and approximate complete genome size in megabase pairs (Mbp) for each *Dadabacteria* MAG. **c** A scatterplot of putative proteome carbon-to-nitrogen content ratio and percent coding density for each *Dadabacteria* MAG. **d** The number of duplicate gene events in each *Dadabacteria* MAG.

As a candidate phylum, a broad understanding of the ecological role of the *Dadabacteria* has remained elusive due to the limited amount of metabolic information available for the clade. Based on the phylogenetic reconstruction of 48 MAGs (mean ± s.d. completeness 75.72% ± 17.77% and contamination 1.85 ± 1.48%; Fig. 1a; Supplementary Table 1), the phylum partitions into three distinct clades which share common environmental features: hydrothermal systems (terrestrial hot springs and hydrothermal vents), organic carbon-associated systems (the terrestrial subsurface, oil-polluted marine systems, marine sponges, marine sediment, and hydrothermal vent sediments), and marine pelagic systems. Within the “marine pelagic” clade, there are two distinct subclades, designated as marine pelagic clade I and II. The marine pelagic clades harbor genomic features that differentiate them from the other clades, specifically with regards to genomic evolutionary selection (e.g., streamlining) and putative metabolisms.

The pelagic marine *Dadabacteria* have undergone a genome streamlining process in comparison to the organic carbon-associated and hydrothermal lineages. The marine pelagic *Dadabacteria* exhibit all five traits associated with genome streamlining: reduced genome size, decreased %GC content, increased C/N ratio in the predicted proteome, increased coding density, and limited/no gene duplication events (Fig. 1b–d; Supplementary Table 1) [6, 13]. The estimated complete marine pelagic *Dadabacteria* genome is ~1.22 Mb (± 0.05 95% CI) with >96% coding density, smaller in size and similar in coding density to the well-studied marine SAR11 clades [8, 60]. The presence of the *Dadabacteria* MAGs reconstructed from multiple oligotrophic *Tara* Oceans regions would suggest that these organisms, like other oligotrophs, are adapted to environments with low nutrient concentrations [6] (Supplementary Fig. 1). Modifications in GC content and proteome C/N ratio are associated with lowering the nitrogen demand for organisms in nitrogen-limited environments [6]. While small genomes, devoid of paralogs and with high coding density, are thought to have reduced energy requirements for division and growth. These genomic modifications which confer an advantage in oligotrophic marine environments are the result of changes in selection pressure that occurred at the transition between the marine pelagic and hydrothermal/organic carbon-associated *Dadabacteria* clades [61, 62]. These results provide further evidence that the theory of genome streamlining is a common evolutionary response to organisms that undergo a transition from nutrient rich to nutrient poor environments [63].

While the SBR1093 MAG was implicated in carbon fixation via the 3-hydroxypriopinate/4-hydroxybutyrate cycle [14], analysis of the *Dadabacteria* phylum reveals, especially for the marine pelagic clades, a predominantly heterotrophic lifestyle (Fig. 1a). Except for the SBR1093 MAG, none of the publicly available *Dadabacteria* MAGs have the potential for carbon fixation (Supplementary Table 2). Several MAGs from the hydrothermal and organic carbon-associated clades have the potential to interface with the nitrogen and sulfur cycles with metabolic processes involved in denitrification, dissimilatory nitrate reduction to ammonia (DNRA), sulfate reduction, sulfide oxidation, and the production of dimethylsulfoniopropionate (DMSP) (Fig. 1a). However, while both marine pelagic clades lack these particular metabolic pathways, all four clades share in the potential for the heterotrophic degradation of proteins and complex carbohydrates, including starch/glycogen (β-glucosidase and α-amylase). One consistent target for the extracellular peptidases (LysM) and carbohydrate-active enzymes (CAZymes; peptidoglycan lyase and CBM Family 50) across the *Dadabacteria* clades is peptidoglycan, the polymer of the microbial cell wall. It may be possible that these predicted proteins are responsible for the internal recycling of the cell wall during cell division or an indication that the *Dadabacteria* occupy a niche capable of recycling microbially derived dissolved organic matter (DOM).

Interestingly, the number of extracellular peptidases, CAZymes, and ATP-binding cassette-type (ABC-type) transporter components normalized for MAG length across all four clades remains consistent even as the overall diversity within each group of proteins decreases (Fig. 1a; Supplementary Tables 3–5). This may highlight an interplay between heterotrophic metabolic diversity and changes in carbon utilization as *Dadabacteria* genome size decreases during streamlining. Additionally, there are several other metabolic processes that distinguish the four clades and highlight the divide between the hydrothermal and organic carbon-associated clades and marine pelagic clades. Specifically, for the hydrothermal clade, the prevalence of CRISPR-associated proteins (used as proxy for CRISPR arrays due low recovery in MAGs), motility, and two-component regulatory chemotaxis suggest that both avoidance of viral predation and physical adjustments within the hydrothermal environment are important evolutionary advantages (Supplementary Tables 2 and 5). Distinct for the hydrothermal and organic carbon-associated clades, are the presence of phosphonate and phosphate ABC transporters, the Entner-Doudoroff pathway, an alternative pathway to glycolysis for glucose degradation, and a Type II secretion system (Supplementary Tables 2 and 6). In many marine systems, phosphorous, like nitrogen, can be a limiting resource. All four clades possess ABC-type phospholipid transporters (Supplementary Table 6), so while most of the marine pelagic clades (63%) lack phosphonate and phosphate transporters, the presence of phospholipid transporters suggest these organisms may recover phosphorous for cellular demand from DOM.

The marine pelagic I and II clades have several distinguishing metabolic properties. Potentially most importantly are the mechanisms related to utilizing light energy. Uniquely amongst the *Dadabacteria*, the marine pelagic I clade possesses rhodopsins and the biosynthetic capacity for retinal synthesis (Fig. 1a). Based on the present amino acids, it is predicted that all of the identified rhodopsins are H^+^-pumping proteorhodopsins [64] (Supplementary Table 7). For the eight identified proteorhodopsins within the marine pelagic I clade, all but one are predicted to be spectrally tuned to absorb blue light [65, 66] (Supplementary Table 7). The marine pelagic I clade also has the capacity to produce terpene secondary metabolites (Supplementary Table 8). Terpenes are organic hydrocarbons that have been shown to be associated with carotenoid synthesis [67]. These terpenes may be related to the production of β-carotene, a biological precursor to retinal, or to production of other unidentified carotenoids (Supplementary Table 6). The marine pelagic II clade lack proteorhodopsins, retinal biosynthesis, and terpene secondary metabolites (Fig. 1a). Like all other *Dadabacteria* clades, the marine pelagic clades possess starch/glycogen and peptidoglycan degradation mechanisms may suggest that these heterotrophic processes are the predominant avenues for energy acquisition.

The metabolic division based on the utilization of light via proteorhodopsins between the marine pelagic clades is reflected in the ecological distribution of the clades. Using a nonredundant set of the marine pelagic *Dadabacteria* MAGs, the large global metagenomic datasets (*Tara* Oceans and bGT) were mapped against the MAGs and used to assess where the *Dadabacteria* occurred through the water column (Supplementary Tables 9 and 10). The two datasets have distinct properties that allow for varying perspectives on the ecology of the *Dadabacteria*. *Tara* Oceans is globally distributed with multiple size fractions and samples from the mesopelagic, while bGT provides several high-resolution cruise tracks with multiple depths between the surface and ~250 m depth. The results from *Tara* Oceans demonstrate that, broadly, the marine clades are present in the planktonic size fraction (<3 μm) and almost exclusively found in the epipelagic (Supplementary Fig. 2).

**Fig. 2:**
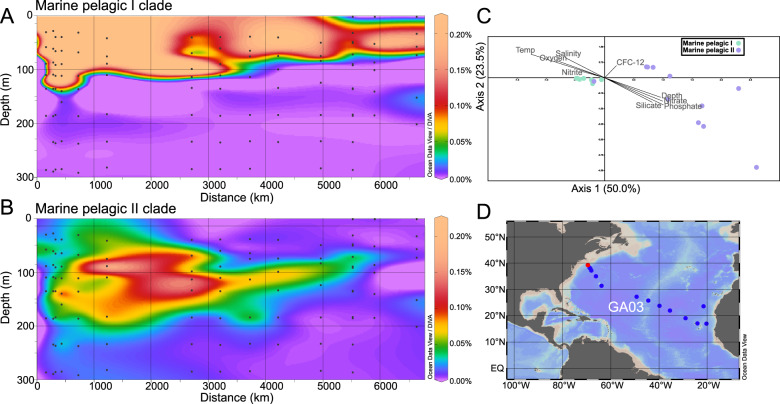
Distribution of the *Dadabacteria* across the North Atlantic. **a** Ocean Data View plot of percent relative fraction for the *Dadabacteria* marine pelagic I clade along the GEOTRACES transect GA03. **b** Ocean Data View plot of percent relative fraction for the *Dadabacteria* marine pelagic II clade along the GEOTRACES transect GA03. **c** Canonical correspondence analysis of the nonredundant marine *Dadabacteria* MAGs. Vectors denote correlations with environmental parameters and have been modified for easier visualization: trioplot amp 1.5, scaling type 2. **d** Cruise track of GA03. Red circle denotes start of cruise track (0 km).

As exemplified by the GA03 cruise track in the North Atlantic, the resolution provided by bGT reveals that the marine pelagic I and II clades tend to be dominant above and below ~100 m depth (~1% light level), respectively, and that this niche transition can be sharp, with the marine pelagic I clade dropping to a negligible component of the microbial community at this partitioning depth (Fig. 2; Supplementary Table 11). This relationship can be observed for the other three cruise tracks, station ALOHA (Hawaii Ocean Time-series), and hydrostation S (Bermuda Atlantic Time-series) with some localized variation, potentially due to surficial mixing and/or downwelling/upwelling events, where the marine pelagic II clade can be found at the surface and the marine pelagic I clade can be found at 250 m. However, for many of the sampling stations there remains a divide between the two clades at the ~1% light depth (Supplementary Figs. 2 and 3). Canonical correspondence analysis (CCA) of the GA03 environmental parameters support this niche transition as a majority of the marine pelagic II clade MAGs correlated with depth and depth-associated parameters (nutrients, temperature, etc.; Fig. 2c). Similar correlations between depth-associated parameters and the marine pelagic clades are observed for the other cruise tracks (Supplementary Fig. 4). As has been shown previously, deep euphotic zone blue-light proteorhodopsins are adapted to low light incidence and capture a limited amount of light at 75 m [68], the apparent depth partitioning linked to encoding proteorhodopsin likely reflects an evolutionary selective pressure against maintaining a light-responsive protein apparatus at depth and manifests as depth-specific niche boundaries between the two marine pelagic clades.

## Conclusion

The *Dadabacteria* phylum is an understudied clade with a limited number of genomic representatives. The broad analysis of the four major clades represented among publicly available genomes reveals a broad range of heterotrophic organisms, putatively involved in the recycling of microbially derived DOM, such as peptidoglycan and phospholipids. The hydrothermal and organic carbon-associated clades appear to be facultative anaerobes capable of using alternative electron acceptors, while the marine pelagic clades appear to be obligate aerobes. The marine pelagic clades have genomic features indicating extensive genome streamlining evolutionary pressures that mirror their ecological distribution in oligotrophic environments. Genome streamlining theory is an important hypothesis for explaining the prevalence of small genomes among cosmopolitan microorganisms and the *Dadabacteria* represent a clear example of the theory in action. The two distinct marine pelagic clades are differentiated in metabolic potential by the presence of light-associated adaptations, such as proteorhodopsin, terpenes, and carotenoids, supporting an argument that marine pelagic I clade possess a photoheterotrophic lifestyle. These adaptations are reflected in the ecological distribution of these clades with depth-partitioned niches for marine pelagic I and II clades. The *Dadabacteria* have multiple transitions that are of interest for understanding evolutionary pressures and adaptations in different environments, including: terrestrial to marine transitions; high to moderate/low temperature transitions; and adaptations from organic rich to organic poor environments. Further studies and the expansion of available genomes for this clade may provide specific insights as to how these transitions occur and manifest in microbial genomes.

## Supplementary information

Supplemental Figures 1-4

Supplemental Table 1

Supplemental Table 2

Supplemental Table 3

Supplemental Table 4

Supplemental Table 5

Supplemental Table 6

Supplemental Table 7

Supplemental Table 8

Supplemental Table 9

Supplemental Table 10

Supplemental Table 11

Supplemental Data 1-4

## Data Availability

Several of the MAGs (TOBG-EAC99, TARA-RED-00009, TOBG-IN994, TOBG-MED731, TOBG-MED713, and TOBG-SP357) used in this study and underwent manual curation originated from the *Tara* Oceans dataset and were never submitted to NCBI to avoid duplication in GenBank. These curated MAGs are noted in Supplementary Table 1 and are available here: 10.6084/m9.figshare.12344207. As noted in Supplementary Table 1, MAGs with corresponding submissions in NCBI GenBank have been updated.
