# Supplementary material for: Marine Dadabacteria exhibit genome streamlining and phototrophy-driven niche partitioning
Source: ISME J. 2020 Nov 23;15(4):1248–56. doi: 10.1038/s41396-020-00834-5 (PMC8115339; doi:10.1038/s41396-020-00834-5)
Supplement: Supplementary file 1 — Supplemental Figures 1-4 [file 41396_2020_834_MOESM1_ESM.pdf]

Supplemental Figures and Tables

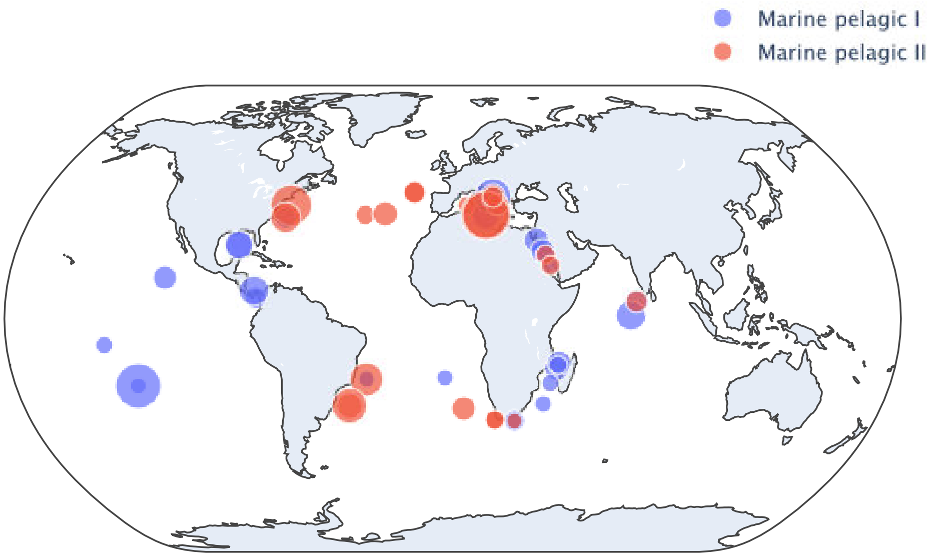

Supplemental Figure 1. Bubble plot of Tara Oceans sites and samples that recruit ≥0.05% relative fraction against the Dadabacteria MAGs. Bubble sizes scale from 0.051-0.471%.

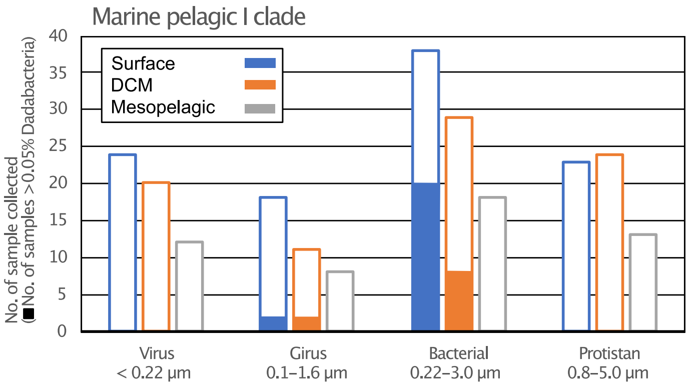

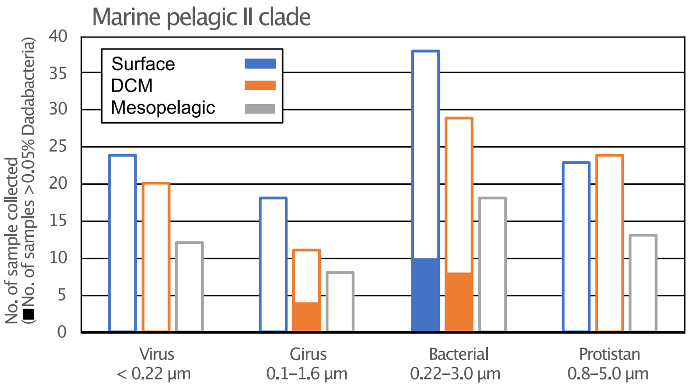

Supplemental Figure 2. Bar plot of the number of Tara Oceans samples from the three depths and four filter fractions targeted as part of the expedition. Bars are filled in according to the number of samples that had ≥0.05% of the metagenome recruit to the MAGs of the marine pelagic I and II clades.

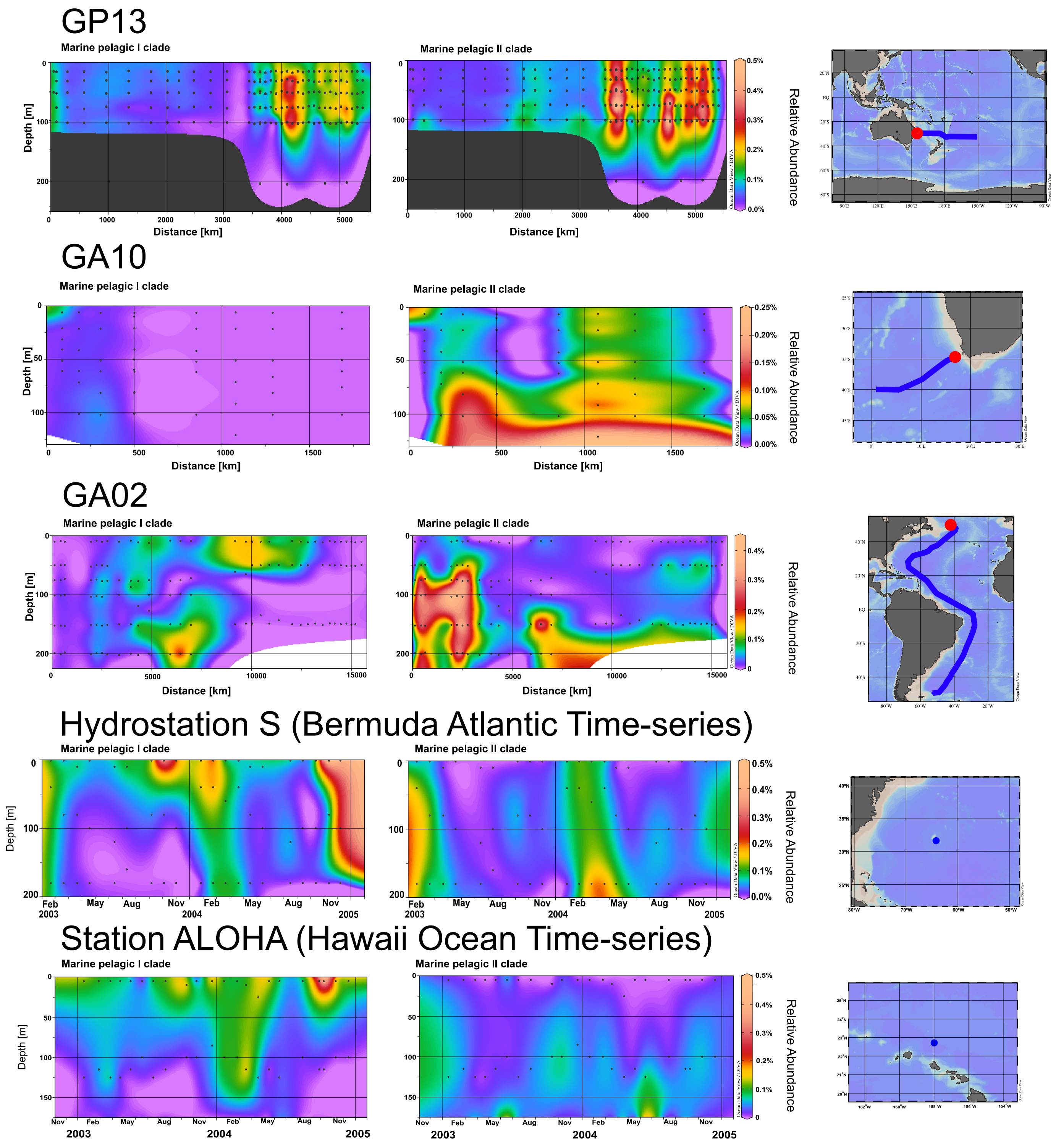

Supplemental Figure 3. Percent relative abundance of the marine pelagic Dadabacteria clades displayed over the length of the three bioGEOTRACES cruise tracks, station ALOHA, and hydrostation S (displayed in the corresponding maps). Red dots denote 0 km marker for each cruise track.

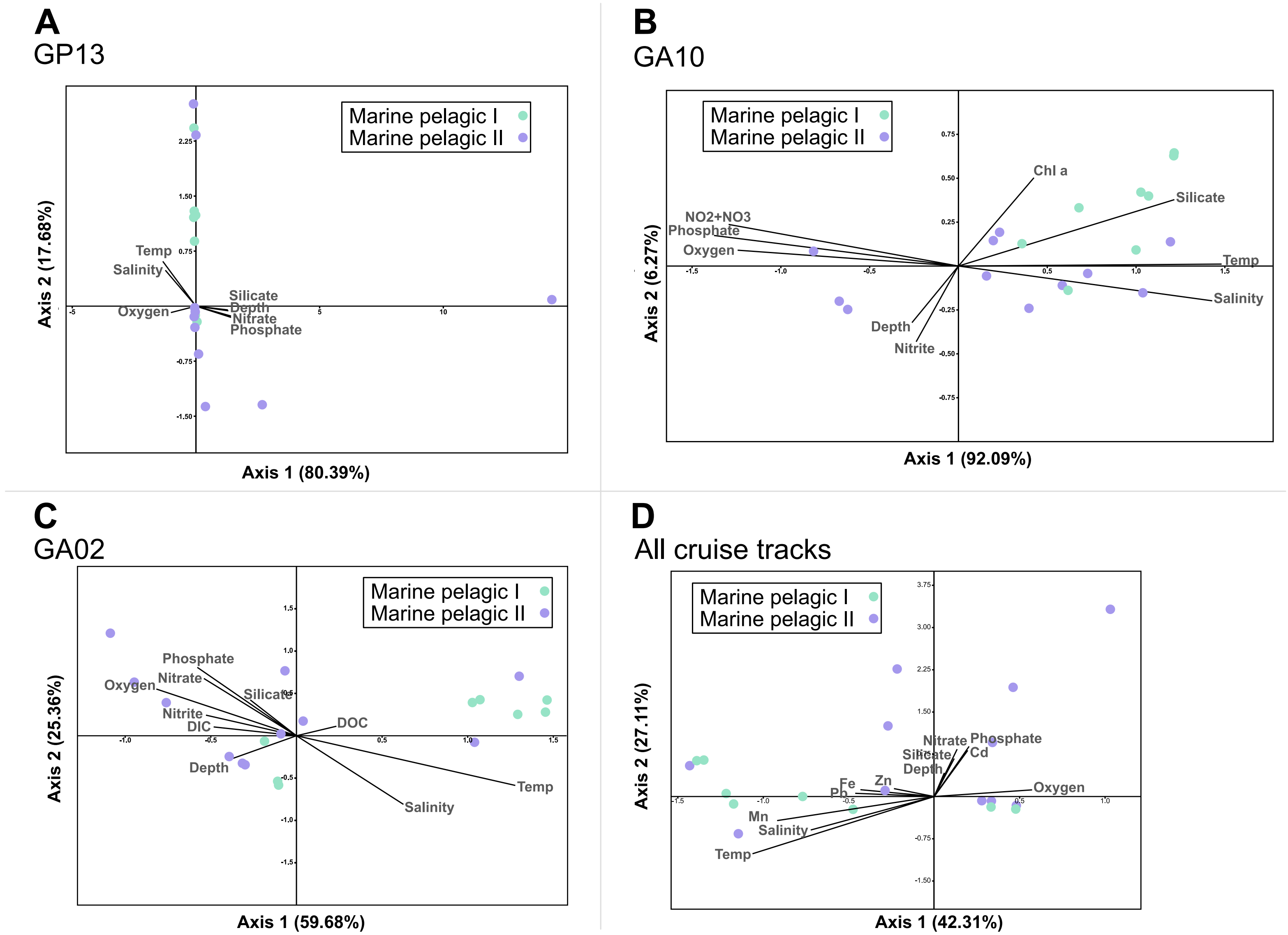

Supplemental Figure 3. Canonical correspondence analysis of marine pelagic Dadabacteria MAGs for three individual bioGEOTRACES cruise tracks and all four cruise tracks combined. Vectors denote correlations with environmental parameters and have been modified for easier visualization: trioplot amp 1.5, scaling type 2.

Table S6. A subset of functions of interest from KEGG and eggNOG determined for each of the four Dadabacteria clades.

	Metabolism	Marine pelagic I	Marine Pelagic II	Hydrothermal	Organic carbon associated
**KEGG**	beta-oxidation (KEGG Module M00087)	✗	✓	✓	✓
	cobalamin (KEGG Module M00122)	✗	✗	?	?
	ABC-type Fe(III) (KEGG KO K02010, K02011, K02012)	✓	✗	✗	✗
	ABC-type heme (KEGG KO K02193, K02194, K02195, K02196)	✓	✓	✗	✗
	ABC-type phospholipid (KEGG KO K02065, K02066, K07122, K02067, K07323)	✓	✓	✓	✓
	ABC-type phosphate (KEGG KO K02036, K02037, K02038, K02040)	✗	✗	✓	✓
	ABC-type branched chain a.a. (KEGG KO K01995, K01996, K01997, K01998, K01999)	✗	✗	✓	✗
	ABC-type lipoprotein (KEGG KO K09810, K09808)	✗	✗	✓	✗
	7,8-dihydro-β-carotene (KEGG KO K10027, K06443)	✓	✗	✗	✗
**NOG (Supplemental Table 5)**	CRISPR associated proteins	✗	✗	✓	✗
	ABC transporter/ABC-type (no. of ABC component types identified - not total count)	**21**	**26**	**59**	**59**
	flagellar	✗	✗	✓	✗
	luciferase	✓	✓	✓	✓
	extracellular ligand-binding	✗	✗	✓	?
	cbb3-type cytochrome	✓	✓	✓	✓
	ammonium transporter	✓	✓	?	✓
	phosphonate transporter	✗	✗	✓	✓

√= present in quorum of MAGs
✗= not present in MAGs
**?** = unclear from genes present if pathway would function

**Table S7. Assignment of detected rhodopsins in the marine pelagic I clade.**							
**Source Genome**	**GeneID**	**MicRhoDE Assigned Class**	**Rhodopsin Class**	**Activity**	**Active Site Amino Acids**	**Spectral Tuning**	**Spectral Tuning Amino Acid**
TOBG-IN994	tobg-in994_270	proteorhodopsin super-cluster IV/Gammaproteobacterial-like cluster 5/SAR86_A-I-like subcluster	proteorhodopsin	proton pump	D97 ; E108	Blue	Q105
TOBG-NAT53	tobg-nat53_530	proteorhodopsin super-cluster IV/Proteobacterial-like cluster 3/HF10_19P19-like subcluster	proteorhodopsin	proton pump	D97 ; E108	Green	L105
TOBG-SP78	sp78_755	proteorhodopsin super-cluster IV/Proteobacterial-like cluster 3/HF10_19P19-like subcluster	proteorhodopsin	proton pump	D97 ; E108	Blue	Q105
TARA-PON-00040	pon00040_717	proteorhodopsin super-cluster IV/Proteobacterial-like cluster 3/HF10_19P19-like subcluster	proteorhodopsin	proton pump	D97 ; E108	Blue	Q105
TARA-RED-00009	red00009_760	proteorhodopsin super-cluster IV/Proteobacterial-like cluster 3/HF10_19P19-like subcluster	proteorhodopsin	proton pump	D97 ; E108	Blue	Q105
TMED126	tmed126_85	proteorhodopsin super-cluster IV/Proteobacterial-like cluster 3/HF10_19P19-like subcluster	proteorhodopsin	proton pump	D97 ; E108	Blue	Q105
TOBG-MED731	tobg-med731_346	proteorhodopsin super-cluster IV/Proteobacterial-like cluster 3/HF10_19P19-like subcluster	proteorhodopsin	proton pump	D97 ; E108	Blue	Q105
TARA-ION-00011	ion00011_392	proteorhodopsin super-cluster IV/Proteobacterial-like cluster 3/HF10_19P19-like subcluster	proteorhodopsin	proton pump	D97 ; E108	Blue	Q105
